# Combination of primary tumor location and mismatch repair status guides adjuvant chemotherapy in stage II colon cancer

**DOI:** 10.18632/oncotarget.21839

**Published:** 2017-10-12

**Authors:** Lin Yang, Wenzhuo He, Qiong Yang, Pengfei Kong, Qiankun Xie, Chang Jiang, Bei Zhang, Liang Ping Xia

**Affiliations:** ^1^ Sun Yat-Sen University Cancer Center, Guangzhou, China; ^2^ State Key Laboratory of Oncology in Southern China, Guangzhou, China; ^3^ Collaborative Innovation Center for Cancer Medicine, Guangzhou, China; ^4^ Sun Yat-Sen Memorial Hospital, Sun Yat-Sen University, Guangzhou, China

**Keywords:** primary tumor location, mismatch repair, survival, adjuvant chemotherapy, colon cancer

## Abstract

**Background:**

Current opinions on the benefits of adjuvant chemotherapy for stage II colon cancer are divided and reformative election of these patients is required. We examined whether the primary tumor location based on mismatch repair status and other risk factors could better inform the current guideline.

**Materials and Methods:**

A total of 673 consecutive patients with stage II colon cancer were included in the analysis. Differences in the common clinicopathological factors between left-sided colon cancer and right-sided colon cancer were analyzed using Fisher's exact analysis. Kaplan–Meier analysis was used to distinguish the survival difference by primary tumor location and/or MMR status.

**Results:**

RCC had a shorter overall survival (*P* = 0.001) and Disease-free survival (*P* = 0.050) than LCC but was associated with survival benefit from adjuvant chemotherapy (*P* = 0.001 and *P* = 0.011 for OS and DFS, respectively). Mismatch repair-proficient had a shorter OS (*P* = 0.036) and disease free survival (*P* = 0.034) than mismatch-repair deficient but chemotherapy improved the OS (*P* = 0.007). When the primary tumor location and MMR status were combined, the PMMR/RCC was the only subgroup that could benefit from adjuvant chemotherapy (*P* < 0.001 and *P* = 0.002 for OS and DFS, respectively). Other tumors such as DMMR/RCC, DMMR/LCC, and PMMR/LCC did not benefit.

**Conclusions:**

The observed survival benefits in PMMR/RCC patients treated with adjuvant chemotherapy will allow better selection of patients for chemotherapy who are in stage II.

## INTRODUCTION

It has been recently reported that colon cancer patients with stage III or high-risk stage II disease treated according to the National Comprehensive Cancer Network (NCCN) guidelines had survival benefit over those patients who received treatment that did not adhere to these guidelines [1]. Based on the NCCN guidelines https://www.nccn.org/ [2], colon cancer patients with low-risk stage II disease can be enrolled in a clinical trial, observed without adjuvant therapy, or considered for capecitabine or 5-FU/leucovorin(LV). For patients with high-risk stage II disease, they can be considered for adjuvant chemotherapy with 5-FU/LV (5-Fluorouracil/Leucovorin), capecitabine, FOLFOX (5-Fluorouracil+oxaliplatin+Leucovorin) CapeOX (Oxaliplatin+Capecitabine), FLOX, or observation. It is a significant challenge to select the most precise option from the aforementioned diversity of choices when a patient with stage II disease presents to healthcare. Firstly, there are numerous risk factors, which are widely accepted as key factors impacting upon the decision to offer adjuvant chemotherapy, then the microsatellite status must also be taken into account. In patients with stage II disease, the medical consensus is that deficiency in mismatched repair protein expression (DMMR) or microsatellite stability high (MSI-H) tumor status are markers of a more favorable outcome and a predictor of decreased benefit (possible a detrimental impact) from adjuvant chemotherapy compared to floropyrimidine alone [3, 4]. In contrast to the aforementioned findings, patients with stage II disease in the QUASAR study showed that although MMR was prognostic, it did not predict benefit or detrimental impact of chemotherapy [5]. A similar conclusion was also reached for patients with stage II disease that were treated with an irinotecan + 5-FU/LV (IFL) regimen [6]. The controversial results limit the value of MSI-H (microsatellite instability-high) in recommending adjuvant chemotherapy or not for patients with stage II disease. Several multigene assays have been developed in the hope of providing prognostic and predictive information in this unusual patient population. However, only the prognostic value has been confirmed [7, 8]

The left and right colon are distinct at both the clinical and molecular level, giving rise to cancers, which have been treated as different diseased since 1990 [9]. Furthermore, the differing characteristics translate into a differential clinical outcome with right colorectal cancer (RCC) presenting a markedly poorer prognosis than left colorectal cancer (LCC) [10, 11]. Recently, the primary tumor location was demonstrated to be predictive of treatment benefit from targeted therapy with anti-EGFR and anti-VEGF agents in metastatic colorectal cancer (mCRC) [12, 13]. This was especially true for cetuximab where survival benefit was only observed in LCC patients [14]. A similar phenomenon was observed for bevacizumab too. After generating a pooled analysis for stage II and III colon cancer patients, Gill [15] found that patients with high-risk resected colon cancer benefited from FU-based therapy only in RCC rather than in LCC. Nevertheless, primary tumor location has only recently been considered a predictor of treatment benefit in association with a limited number of targeted agents in mCRC. The evidence to date has not yet confirmed that primary tumor location can help the choice of adjuvant chemotherapy in patients with stage II disease alone, or combined with MMR status. Therefore, this is the main purpose of this study.

## MATERIALS AND METHODS

We retrospectively studied a total of 673 eligible patients who received radical surgical resection for colorectal cancer treated at Sun Yat-sen University Cancer Center between October 2004 and March 2014. The inclusion criteria for the study were as follows: (i) pathological evidence of CRC; (ii) complete baseline clinical information and laboratory data; (iii) clinical stage II according to the American Joint Commission on Cancer/International Union Against Cancer (AJCC/UICC, the seventh version) and (iv) complete follow-up data. The exclusion criteria included (i) patients with rectal cancer; (ii) patients with ascertained MMR status; (iii) patients with more than one primary tumor that was concurrent; (iv) patients who died of non-cancer related diseased. Ethical approval was obtained from both institutions through the respective institutional review boards. The study protocol was designed in accordance with the guidelines outlined in the Declaration of Helsinki and was approved by the Ethics Committee of Sun Yat-sen University Cancer Center.

Colon cancers located in the cecum, ascending colon, and transverse colon were defined as RCC, while those located in the descending or sigmoid colon were defined as LCC [16–20]. Overall survival (OS) was defined as the time from the date of initial diagnosis to the date of death from cancer-related cause or until the date of the last follow-up. Disease-free survival (DFS) was ruled as the time from radical surgery time to the date diagnosed with the distant metastasis or relapse, death from cancer-related cause, or until the date of the last follow-up. The median follow-up time was 111 months (range: 4–151 months) for OS and 105 months (range: 4–132 months) for DFS.

### Assessment of the CEA, CA199, and CRP

All samples were collected before any treatment and were tested within 24 h after collection. The supernatants were processed for analysis of CEA and CA199 on a UniCelDxI 800 immunoassay system (Beckman Coulter, Brea, CA). Plasma CRP was measured using a high-sensitivity assay (Beckman Coulter, Woerden, the Netherlands) as previously described [21].

### MMR status determination

The four most common mismatch repair proteins were assessed by immunohistochemistry using the standard Envision two-step procedure. Briefly, the slides were backed at 60°C for 2 hours, cleared through xylene, rehydrated, pre-treated with EDTA antigen retrieval buffer, treated in 3% hydrogen for 20 min to prevent endogenous peroxidase activities, and then incubated with 10% normal goat serum at 37°C to block non-specific activity. Then, the slides were incubated at 4°C overnight using the following antibodies: MLH1 (1:50; Beijing Zhong Shan -Golden Bridge Biological Technology, Beijing, China), PMS2 (1:50; Beijing Zhong Shan -Golden Bridge Biological Technology, Beijing, China), MSH2 (1:50; Beijing Zhong Shan -Golden Bridge Biological Technology, Beijing, China) and MSH6 (1:50; Beijing Zhong Shan -Golden Bridge Biological Technology, Beijing, China). The tissues were incubated with a secondary antibody after washing (Envision; Dako, Glostrup, Denmark) for 1 hour at room temperature. Finally, the sections were systematically counterstained with 10% Mayer's hematoxylin, before dehydration and mounting in Crystal Mount. The known MMR-deficient colorectal carcinomas were observed as external negative controls and the non-neoplastic colonic mucosa, stromal cells, infiltrating lymphocytes or the centers of lymphoid follicles were regarded as internal positive controls. Immunostaining scores were recorded by two experienced pathologists and without prior knowledge of the patients’ clinical data. Nuclear staining within tumor cells was defined as normal expression, while complete absence of nuclear staining within tumor cells with concurrent internal positive controls was considered to be negative protein expression. MLH1/PMS2/MSH2/MSH6 protein expression negative cells were defined as tumors with loss of MLH1/PMS2/MSH2/MSH6 protein, visualized by light microscopy.

### Statistical analysis

The continuous variables were transformed into dichotomous variables and the median value was used. The threshold of CEA and CA-199 were established at 5 ng/ml and 37 U/ml as commonly suggested [22]. Comparisons were performed using Fisher's exact test. The Kaplan–Meier method was used to calculate the OS and DFS survival curves, and the difference was evaluated using the log-rank test. Statistical analyses of survival data were performed using SPSS for Windows, version 19.0 (SPSS, Chicago, IL, USA). Two-sided *P* values < 0.05 were deemed significant. All data has been deposited at Sun Yat-sen University Cancer Center for future reference (RDD number: RDDA2017000269).

## RESULTS

### Patient characteristics and survival

**Figure 1:**
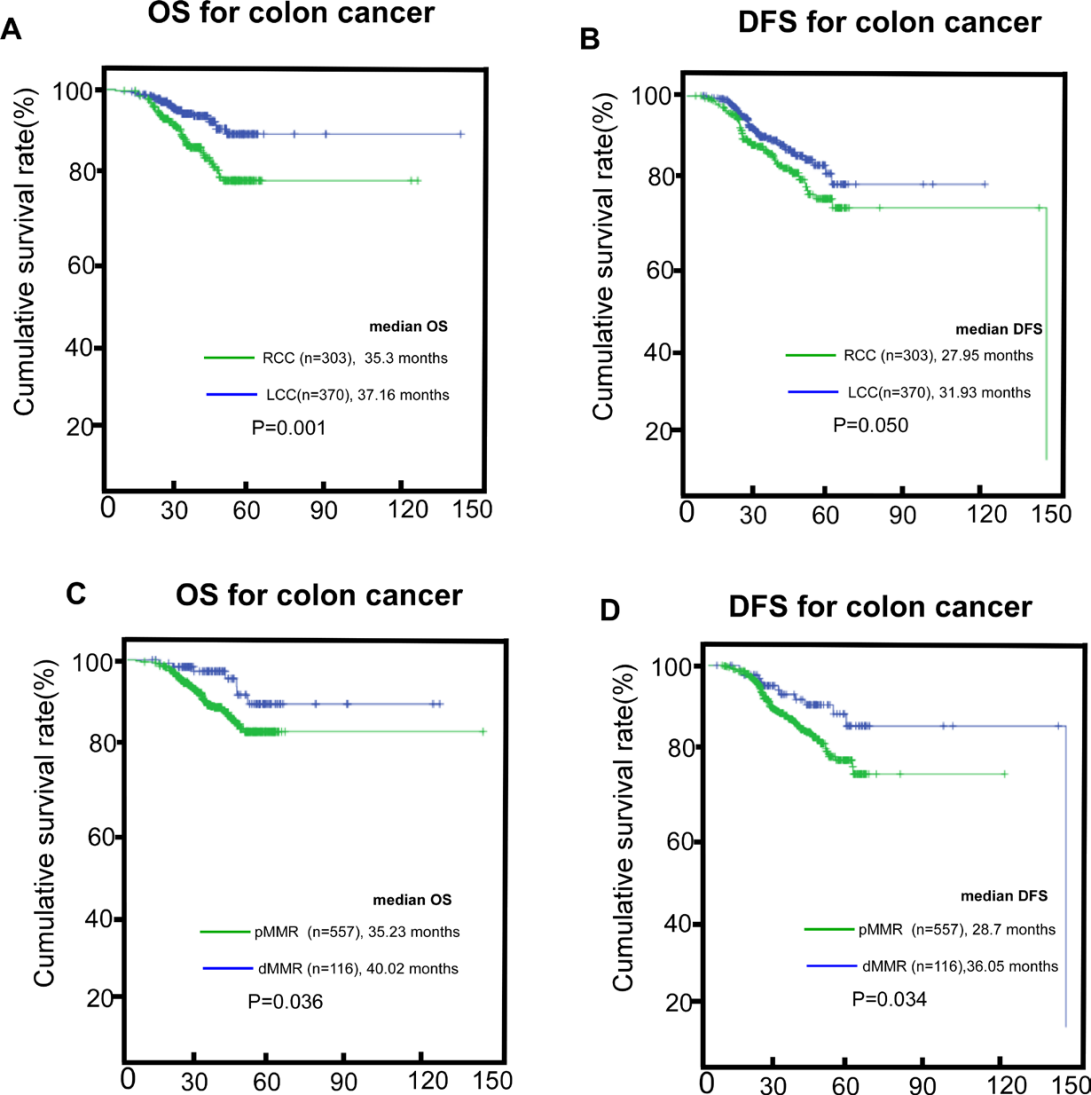
The survival (OS, overall survival; DFS, disease-free survival) difference in RCC (right-sided colon cancer) and LCC (left-sided colon cancer) in the whole cohort, (**A** and **B**) the survival difference between the dMMR (mismatch-repair deficient) cohort and the pMMR (mismatch-repair proficient) cohort, (**C** and **D**).

A total of 673 patients with stage II CRC were enrolled, including 370 patients with LCC and 303 patients with RCC. At the end of the study period (March, 2017), 74 (11.0%) patients had died because of cancer-related disease and 105 (15.6%) patients had distant metastasis or recurrence. The 5-year OS rate for patients with LCC was 83.5% compared with 77.3% for patients with RCC (*P* = 0.001, Figure 1A). There was also an apparently poorer DFS for those with RCC (*P* = 0.050, Figure 1B). The dMMR cohort also had improved survival compared with the pMMR cohort (*P* = 0.036 and *P* = 0.034 for OS and DFS, respectively, Figure 1C, 1D). The 5-year survival rate was 87% for the dMMR cohort, whereas it was merely 79.0% for the pMMR cohort. All common clinicopathologic features between RCC and LCC, and between dMMR and pMMR are shown in Table 1. Those with RCC tended to have higher CRP values (*P* < 0.001), lower ALB (*P* < 0.001), adenocarcinoma, (*P* < 0.001), pMMR (*P* < 0.001), and TLN >12 (*P* < 0.001). Furthermore, the pMMR cohort was older (*P* = 0.032), had higher CR*P* values (*P* < 0.001), higher ALB (albumin) (*P* = 0.005), non-adenocarcinoma (*P* = 0.010), advanced stage cancer (*P* = 0.004), had RCC primary tumors (*P* < 0.001), TLN (Total lymph node) > 12 (*P* < 0.001), vascular invasion (*P* = 0.030), and had been treated with chemotherapy (*P* = 0.001).

**Table 1:** The clinicopathogical characteristics of RCC and LCC groups, and dMMR and pMMR groups

Characteristics	All, Number (%)	LCC, Number (%)	RCC, Number (%)	*P*	dMMR, Number (%)	pMMR, Number (%)	*P*
**Age, years**				**0.080**			**0.032**
< 59	357 (53.0%)	185 (51.8%)	172 (48.2%)		72 (20.2%)	285 (79.8%)	
≥ 59	316 (47.0%)	185 (58.5%)	131 (41.5%)		44 (13.9%)	272 (86.1%)	
**Sex**				**0.077**			**0.076**
Male	409 (60.8%)	236 (57.7%)	173 (42.3%)		62 (15.2%)	347 (84.8%)	
Female	264 (39.2%)	134 (50.8%)	130 (49.2%)		54 (20.5%)	210 (79.5%)	
**CRP, mg/L**				**< 0.001**			< 0.001
< 3.26	333 (49.5%)	210 (63.1%)	123 (36.9%)		36 (10.8%)	297 (89.2%)	
≥ 3.26	340 (50.5%)	160 (47.1%)	180 (52.9%)		80 (23.5%)	260 (76.5%)	
**ALB, g/L**				**< 0.001**			**0.005**
< 40.2	367 (54.5%)	177 (48.2%)	190 (51.8%)		77 (21.0%)	290 (79.0%)	
≥ 40.2	306 (45.5%)	193 (63.1%)	113 (36.9%)		39 (12.7%)	267 (87.3%)	
**CEA, ng/mL**				0.204			0.276
< 5	447 (66.4%)	238 (53.2%)	209 (46.8%)		72 (16.1%)	375 (83.9%)	
≥ 5	226 (33.6%)	132 (58.4%)	94 (41.6%)		44 (19.5%)	182 (80.5%)	
**CA199, U/mL**				**0.394**			0.235
< 37	575 (85.4%)	320 (55.7%)	255 (44.3%)		95 (16.5%)	480 (83.5%)	
≥ 37	98 (14.6%)	50 (51.0%)	48 (49.0%)		21 (21.4%)	77 (78.6%)	
**Pathology**				**< 0.001**			**0.010**
Adenocarcinoma	579 (86.0%)	340 (58.7%)	239 (41.3%)		25 (26.6%)	69 (73.4%)	
Mucinous or signet-ring cell	94 (14.05)	30 (31.9%)	64 (68.1%)		91 (15.7%)	488 (84.3%)	
**Tumor location**							**< 0.001**
LCC	370 (55.0%)				36 (9.7%)	334 (90.3%)	
RCC	303 (44.9%)				80 (26.4%)	223 (73.6%)	
**MMR**				**< 0.001**			
dMMR	116 (17.2%)	36 (31.0%)	80 (69.0%)				
pMMR	557 (82.8%)	334 (60.0%)	223 (73.6%)				
**T stage**				**0.350**			**0.004**
IIa	539 (80.1%)	302 (56.0%)	237 (44.0%)		81 (15.0%)	458 (85.0%)	
IIb	106 (15.8%)	56 (52.8%)	50 (47.2%)		30 (28.3%)	76 (71.7%)	
IIc	28 (4.2%)	12 (42.9%)	16 (57.1%)		5 (17.9%)	23 (82.1%)	
**TLN**				< 0.001			**0.014**
< 12	247 (36.7%)	184 (74.5%)	63 (25.5%)		31 (12.6%)	216 (87.4%)	
≥ 12	426 (63.3%)	186 (43.7%)	240 (56.3%)		85 (20.0%)	341 (80.0%)	
**Vascular invasion**				0.437			0.030
No	585 (86.9%)	325 (55.6%)	260 (44.4%)		108 (18.5%)	477 (81.5%)	
Yes	88 (13.1%)	45 (51.1%)	43 (48.9%)		8 (9.1%)	80 (90.9%)%)	
**Nerve invasion**				0.667			0.194
No	665 (98.8)	365 (54.9%)	300 (45.1%)		116 (17.4%)	549 (82.6%)	
Yes	8 (1.2%)	5 (62.5%)	3 (37.5%)		0 (0.0%)	8 (100.0%)	
**Intestinal obstruction**				0.284			0.262
No	667 (99.1%)	368 (55.2%)	299 (44.8%)		116 (17.4%)	551 (82.6%)	
Yes	6 (0.9%)	2 (33.3%)	4 (66.7%)		0 (0.0%)	6 (100.0%)	
**Chemotherapy**				0.477			0.001
Yes	243 (36.1%)	138 (56.8%)	105 (43.2%)		26 (10.7%)	217 (89.3%)	
No	430 (63.9%)	232 (54.0%)	198 (46.0%)		90 (20.9%)	340 (79.1%)	
**Survival status**				< 0.001			0.060
Live	599 (89.0%)	**345 (57.6%)**	254 (42.4%)		109 (18.2%)	490 (81.8%)	
Dead	74 (11.0%)	**25 (33.8%)**	49 (66.2%)		7 (9.5%)	67 (90.5%)	
**Distant metastasis or relapse**				0.007			0.152
Yes	105 (15.6%)	45 (42.9%)	60 (57.1%)		13 (12.4%)	92 (87.6%)	
No	568 (84.4%)	325 (57.2%)	243 (42.8%)		103 (18.1%)	465 (81.9%)	

Abbreviations: CRC, colon cancer; LCC, Left-sided colon cancer; RCC, right-sided colon cancer; CRP, C-reactive protein; WBCs, white blood cells; ALB, albumin; CA199, Carbohydrate Antigen 19–9; CEA, Carcinoembryonic antigen; MMR, Mismatch repair; dMMR, mismatch-repair deficient; pMMR, mismatch-repair proficient; TLN, Total lymph node cleared in surgery.

### The adjuvant chemotherapy benefit with primary tumor location

**Figure 2:**
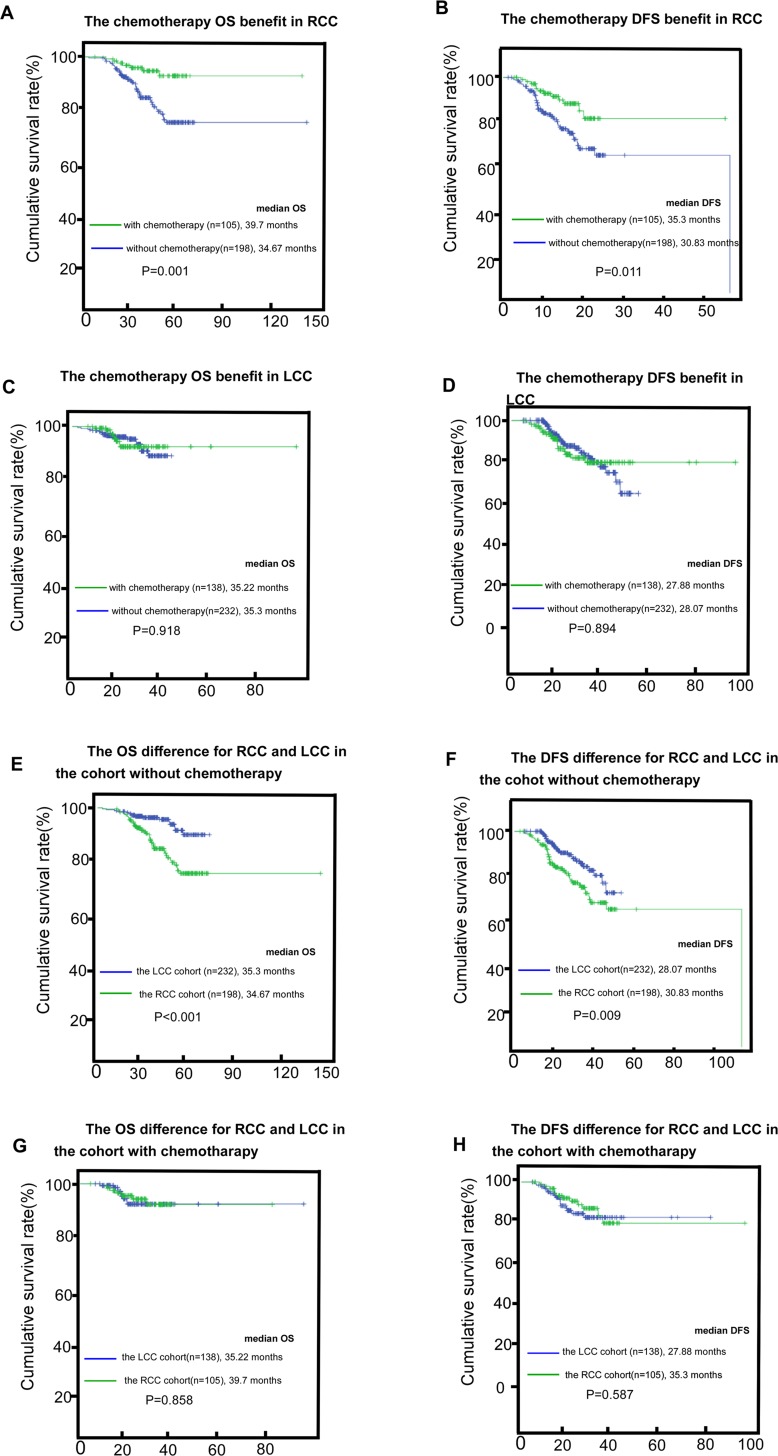
The chemotherapy benefit in RCC (right-sided colon cancer) and LCC (left-sided colon cancer) for OS (overall survival) and DFS (disease-free survival), (**A**–**D**) the survival (OS and DFS) difference between RCC and LCC in the subgroups without adjuvant-chemotherapy or with adjuvant-chemotherapy, (**E**–**H**).

The chemotherapy benefit was observed in the RCC cohort (*P* = 0.001 and *P* = 0.011 for OS and DFS, respectively, Figure 2A, 2B). However, it was not observed in the LCC cohort (*P* = 0.918 and *P* = 0.894 for OS and DFS, respectively; Figure 2C, 2D). Those with LCC had apparently better survival than the RCC cohort in the non-chemotherapy group (*P* < 0.001 and *P* = 0.009 for OS and DFS, respectively; Figure 2E, 2F). The improved LCC survival benefit over the RCC cohort disappeared in the chemotherapy group (*P* = 0.858 and *P* = 0.587 for OS and DFS, respectively; Figure 2G, 2H).

### The adjuvant chemotherapy benefit with mismatch repair status

**Figure 3:**
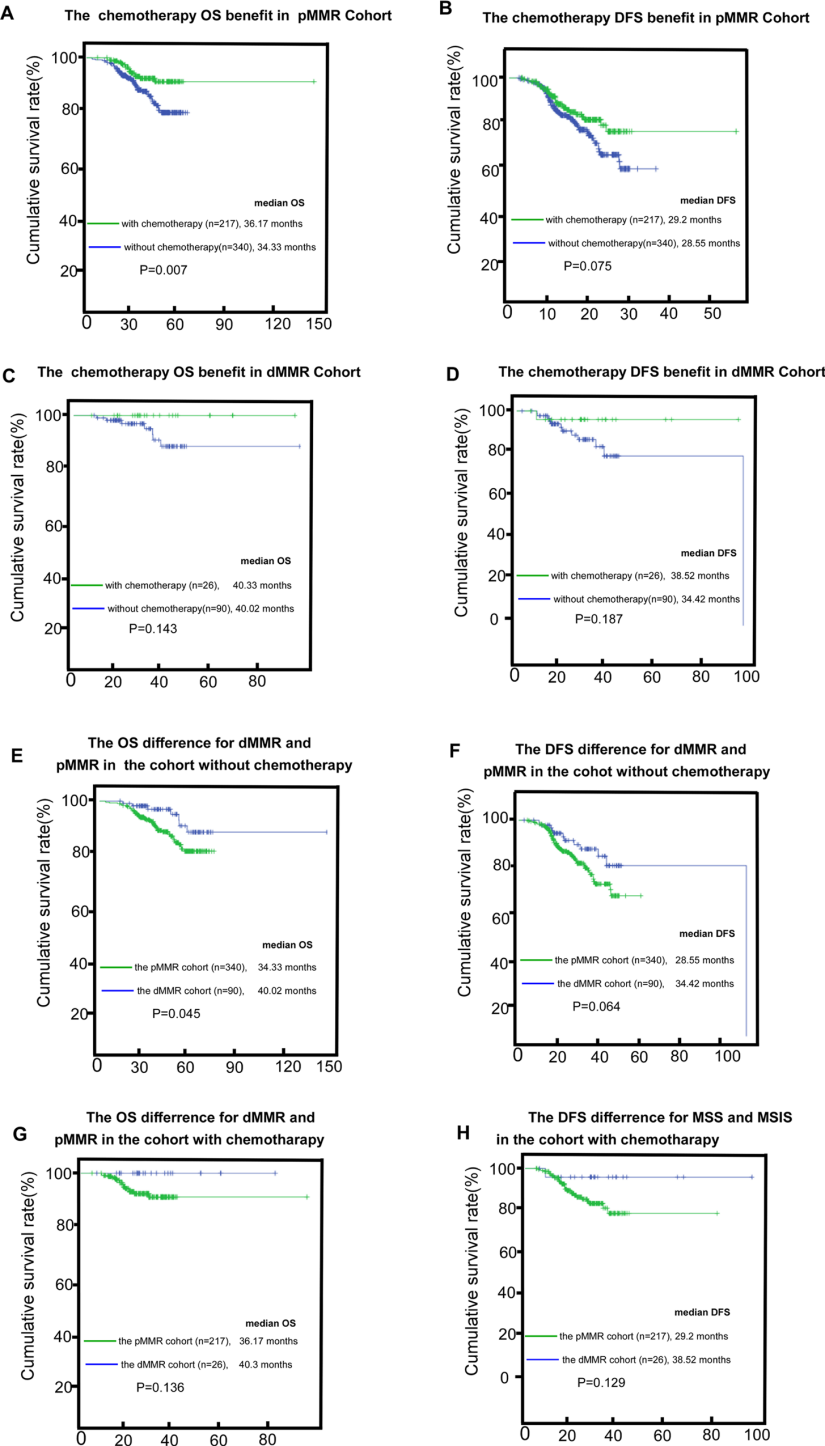
The chemotherapy benefit in the dMMR (mismatch-repair deficient) and the pMMR (mismatch-repair proficient) for OS (overall survival) and DFS (disease-free survival), (**A**–**D**); the survival (OS and DFS) difference between dMMR and pMMR in the subgroups without adjuvant-chemotherapy or with adjuvant-chemotherapy, (**E**–**H**).

The pMMR cohort had an improved OS rather than improved DFS if they received adjuvant chemotherapy (*P* = 0.007 and *P* = 0.075 for OS and DFS, respectively; Figure 3A, 3B). The dMMR cohort that received adjuvant chemotherapy did not attain survival benefit (*P* = 0.143 and *P* = 0.187 for OS and DFS, respectively; Figure 3C, 3D). As expected, in the non-chemotherapy cohort, the dMMR cohort had a better prognosis than the pMMR cohort for OS (*P* = 0.045, Figure 3E) but a significant difference in DFS was not observed (*P* = 0.064, Figure 3F) while the difference narrowed even further after chemotherapy (*P* = 0.136 and *P* = 0.129, respectively; Figure 3G and 3H).

### The combination of the MMR/primary tumor location survival analysis

**Figure 4:**
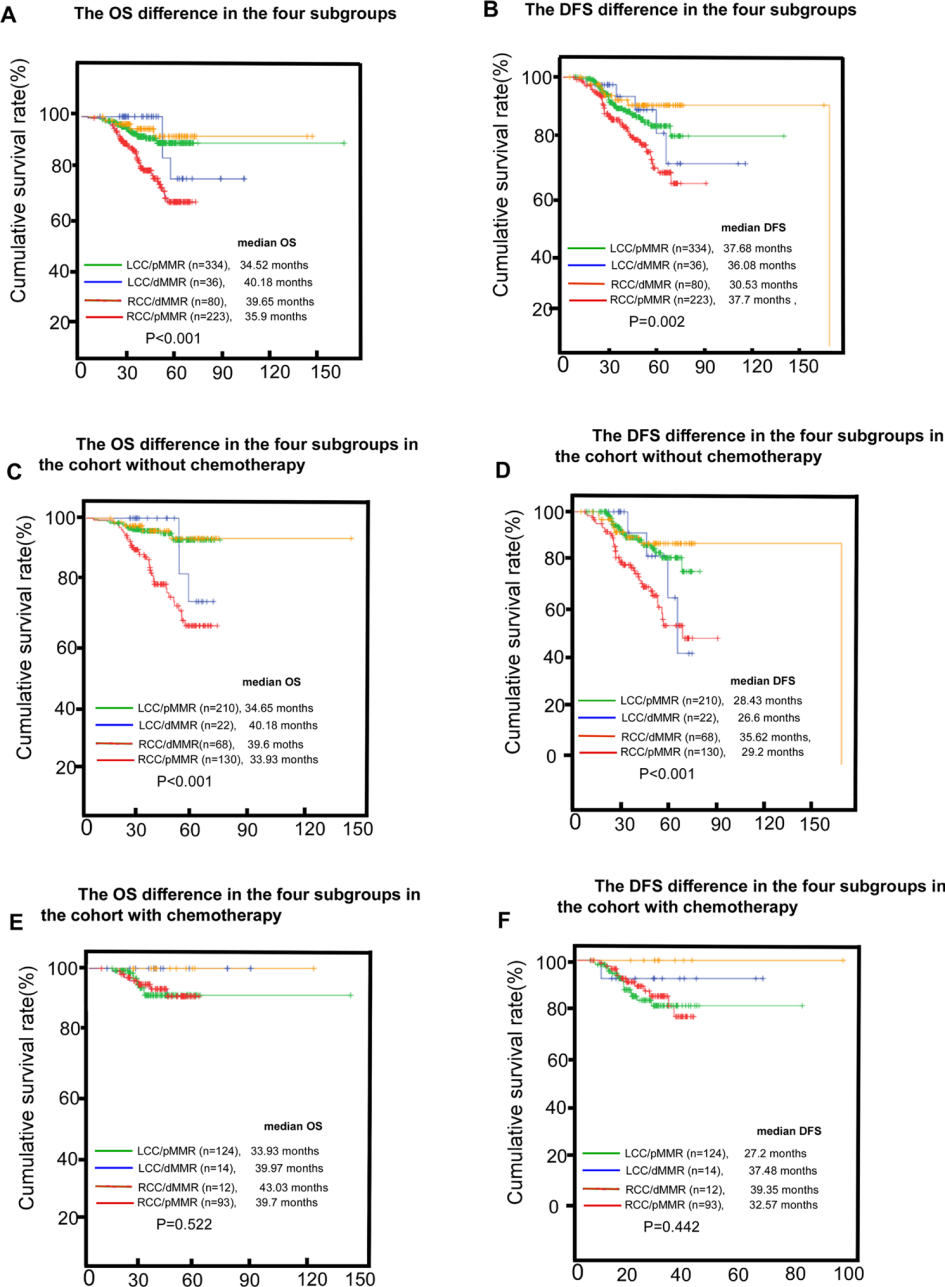
The survival (OS, overall survival; DFS, disease-free survival) difference between the four subgroups (RCC/pMMR, right-sided colon cancer/mismatch-repair proficient, RCC/dMMR, right-sided colon cancer/mismatch-repair deficient, LCC/dMMR, left-sided colon cancer/mismatch-repair deficient, LCC/pMMR, left-sided colon cancer/ mismatch-repair proficient) in the whole cohort (**A**, **B**), the cohort without chemotherapy (**C**, **D**) or the cohort without chemotherapy (**E**, **F**).

**Figure 5:**
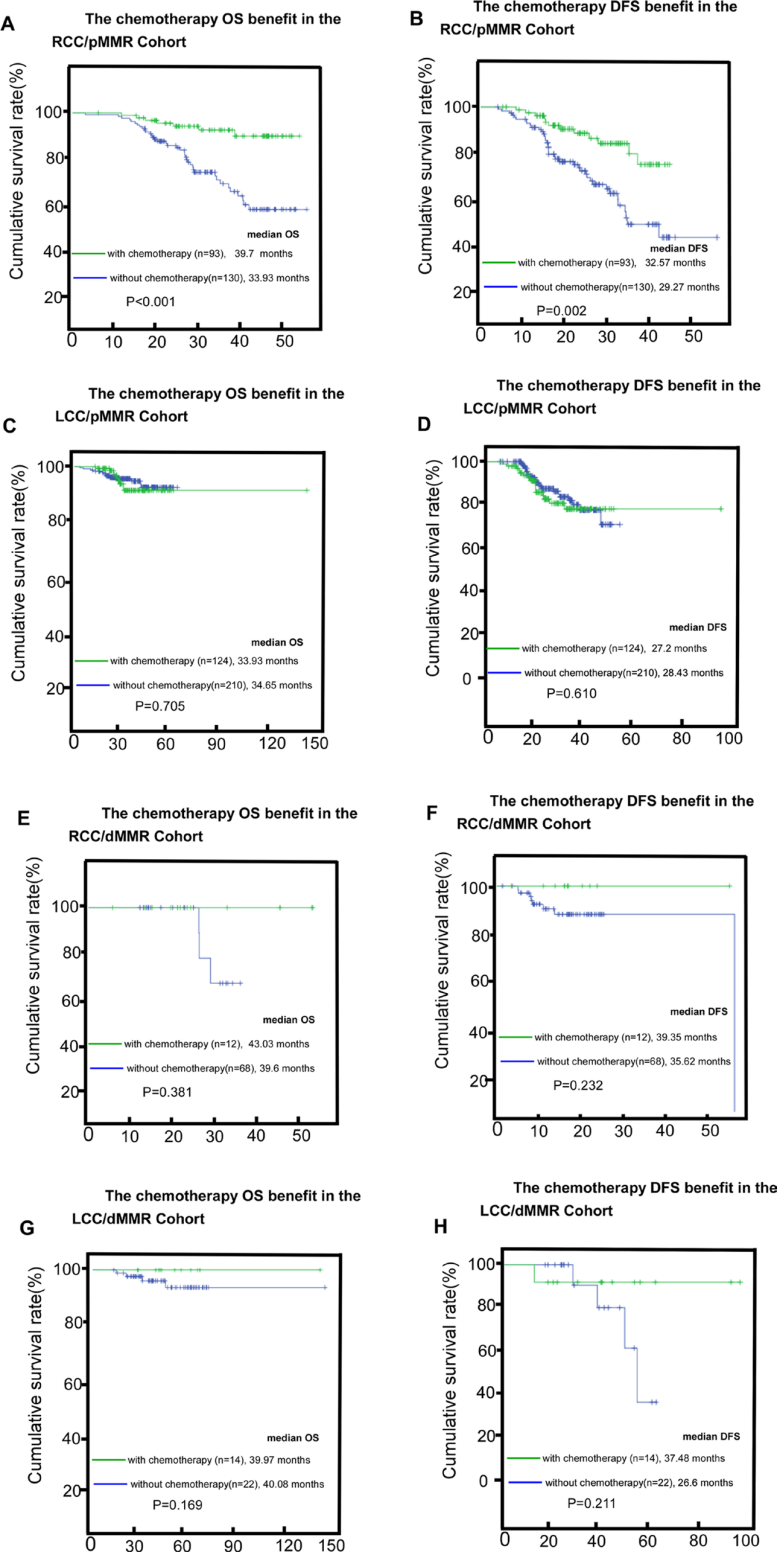
The adjuvant-chemotherapy survival (OS, overall survival, DFS, disease-free survival) benefit in the four subgroups (RCC/pMMR, right-sided colon cancer/ mismatch-repair proficient (**A**, **B**), LCC/pMMR, left-sided colon cancer/ mismatch-repair proficient (**C**, **D**), RCC/dMMR, right-sided colon cancer/ mismatch-repair deficient (**E**, **F**), LCC/dMMR, left-sided colon cancer/ mismatch-repair deficient (**G**, **H**).

We classified CRCs into four groups based on MMR status and primary tumor location: pMMR /RCC, dMMR /RCC, pMMR /LCC, and dMMR /LCC. We looked at both the whole cohort and the non-chemotherapy cohort, and compared the pMMR /RCC cancers. We found that the dMMR /RCC, dMMR /LCC, and pMMR /LCC cases exhibited significantly better outcomes (*P* < 0.001 and *P* < 0.001 for OS, *P* = 0.002 and *P* < 0.001 for DFS, Figure 4A–4D). The survival differences disappeared in the chemotherapy cohort (*P* = 0.522 and *P* = 0.442 for OS and DFS, respectively; Figure 4E, 4F). The pMMR/RCC cohort gained significant benefit with chemotherapy (*P* < 0.001 and *P* = 0.002 for OS and DFS, respectively; Figure 5A, 5B), whereas pMMR/LCC, dMMR/RCC, dMMR/LCC or other subgroups derived no benefit (*P* = 0.705, *P* = 0.381 and *P* = 0.169 for OS; *P* = 0.610, *P* = 0.232 and *P* = 0.211 for DFS, respectively; Figure 5C–5H).

We analyzed the distribution of different chemotherapeutic regimens in the four subgroups, as shown in Table 2 but these were not significantly different (*P* = 0.986). 430 patients without adjuvant chemotherapy and 243 patients were recommended to follow the 5-FU-based adjuvant-chemotherapy regimens. This included 1 patient with 5-Fu/LV, 98 patients with Xeloda, 136 patients with XELOX, and 8 patients with FOLFOX.

**Table 2:** The distribution of chemotherapeutic regimens among the four subgroups after combining primary tumor location and mismatch repair status

Characteristics	All, Number (%)	Single-chemotherapy, Number (%)	Combined-chemotherapy, Number (%)	*P*
**MMR status/primary tumor location**				0.986
dMMR/LCC	14 (4.7%)	4 (28.6%)	10 (71.4%)	
pMMR/LCC	158 (53.0%)	53 (33.5%)	105 (66.5%)	
dMMR/RCC	21 (7.0%)	7 (33.3%)	14 (66.7%)	
pMMR/RCC	105 (35.2%)	35 (33.3%)	70 (66.7%)	

Abbreviations: dMMR/LCC, mismatch-repair deficient/left-sided colon cancer; pMMR/LCC, mismatch-repair proficient/left-sided colon cancer; dMMR/RCC, mismatch-repair deficient/right-sided colon cancer; pMMR/RCC, mismatch-repair proficient/right-sided colon cancer.

Furthermore, we compared the number of risk factors, MMR status, TLN number, T stage, vascular invasion, nerve invasion, intestinal obstruction, and intestinal perforation, between the chemotherapy cohorts and non-chemotherapy cohort for the four groups. As shown in Table 3, the number of risk factors balanced across all groups (*P* = 0.250, *P* = 0.684, *P* = 0.547, and *P* = 0.530, respectively; Table 3).

**Table 3:** The distribution of the number of risk factors after combining primary tumor location and mismatch repair status among the four subgroups respectively

Characteristics	All, Number (%)	Non-chemotherapy, Number (%)	With chemotherapy, Number (%)	*P*
dMMR/LCC^*^				
** The number of risk factors**				0.250
0	12 (33.3%)	4 (33.3%)	8 (66.7%)	
1	18 (50.0%)	3 (16.7%)	15 (83.3%)	
2	6 (16.7%)	3 (50.0%)	3 (50.0%)	
pMMR/LCC^**^				
** The number of risk factors**				0.684
0	114 (34.1%)	74 (64.9%)	40 (35.1%)	
1	169 (50.6%)	102 (60.4%)	67 (39.6%)	
2	47 (14.1%)	32 (68.1%)	15 (31.9%)	
3	4 (1.2%)	2 (50.0%)	2 (50.0%)	
dMMR/RCC^***^				
** The number of risk factors**				0.547
0	9 (11.3%)	8 (88.9%)	1 (11.1%)	
1	45 (56.3%)	36 (80.0%)	9 (20.0%)	
2	25 (31.3%)	23 (92.0%)	2 (8.0%)	
3	1 (1.3%)	1 (100.0%)	0 (0.0%)	
pMMR/ RCC^****^				
** The number of risk factors**				0.530
0	29 (13.0%)	19 (65.5%)	10 (34.5%)	
1	135 (60.5%)	75 (55.6%)	60 (44.4%)	
2	54 (24.2%)	34 (63.0%)	20 (37.0%)	
3	5 (2.2%)	2 (40.0%)	3 (60.0%)	

Abbreviations: dMMR/LCC, mismatch-repair deficient/left-sided colon cancer; pMMR/LCC, mismatch-repair proficient/left-sided colon cancer; dMMR/RCC, mismatch-repair deficient/right-sided colon cancer; pMMR/RCC, mismatch-repair proficient/right-sided colon cancer.

**Figure 6:**
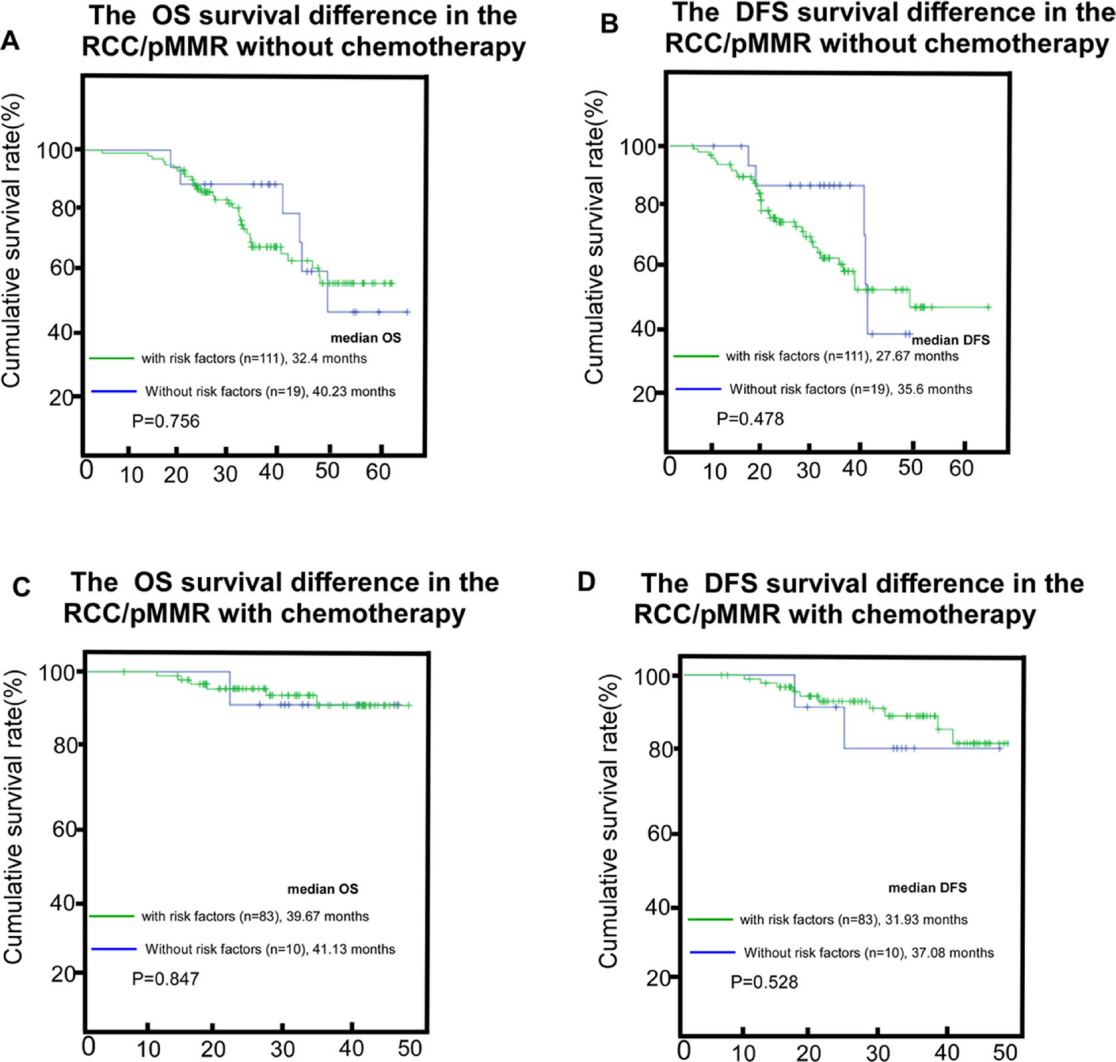
The survival difference between the subgroups with the risk-factors and the subgroups without risk-factors in the RCC/pMMR cohort without adjuvant-chemotherapy, (**A** and **B**) the survival difference between the subgroups with the risk-factors and the subgroups without risk-factors in the RCC/pMMR cohort with adjuvant-chemotherapy, (**C** and **D**) risk factors include one of the following factors: perforated cancer, pT4N0 with vascular emboli, vascular invasion and/or obstructive colorectal tumor; RCC/pMMR, right-sided colon cancer/ mismatch-repair proficient.

Additionally, in the pMMR/RCC subgroup receiving adjuvant chemotherapy, there was no survival difference between the cohorts with or without the risk factors (*P* = 0.756 for OS and *P* = 0.478 for DFS, Figure 6). Similar results were found in the pMMR/RCC subgroup without adjuvant chemotherapy (*P* = 0.847 for OS and *P* = 0.528 for DFS, Figure 6).

## DISCUSSION

In mCRC [12, 23] or CRC without distinguished stages [24], the primary tumor location is a prognostic factor that suggests those with LCC have a significantly longer OS than RCC. However, its prognostic value has not been fully studied in these early studies. Our results showed that those with LCC had survival advantages compared with RCC patients, and that these were evident for stage II disease too. Similar findings in our previous study exploring mCRC were also noted [25]. Both OS and DFS advantages associated with LCC were not only evident for all stage II disease but also existed in patients without chemotherapy, which excluded the influence of treatment interventions. It is also worth noting that the common prognostic clinicopathological features were very similar between LCC and RCC patients. These data strongly support the poor prognosis of RCC in stage II disease. The same comparison was conducted in dMMR and pMMR, where the results showed that those with dMMR had a much longer OS than pMMR, which is in accordance with other studies [26, 27].

The role of MMR status guiding adjuvant chemotherapy in stage II CRC was studied, and the results accord with references that suggest MSI patients are unable to obtain survival benefit from chemotherapy [26]. The primary tumor location as a predictor of targeted agents in mCRC has been fully explored in recent years and showed that cetuximab survival benefit was limited to LCC patients [13, 28–30]. However, while the majority of studies found that only LCC patients gained survival advantages from bevacizumab [12, 25, 31], some studies found that those CRC in both sides can get survival benefit, although LCC gained more [32]. Nevertheless, the predictor value of this parameter was not fully investigated in the early stages of disease. We found that the chemotherapy survival benefit was confined only to those with RCC, which differs from studies using targeted agents. The causes for this are unclear but may be due partly to the good response in the MSI tumor subgroup, comprising 20% of cases at this site [17, 33–36]. In addition, more men tend to have distributed cancer in the left hand side [37], LCCs are more common in high-incidence regions [38], and geographical and sex-related differences in the incidence of colorectal cancer may be attributable to dietary and hormonal or reproductive factors, respectively. Randomized trials have also shown survival benefits from chemotherapy in rectal cancer [39].

Current opinion on the benefits of adjuvant chemotherapy for stage II tumors is divided [40, 41]. Although risk factors can indicate success of adjuvant chemotherapy in stage II disease, many questions persist e.g. is the weight of each risk factor equal? Furthermore, what is the different impact on survival time or chemotherapy benefit when patients have one or more risk factor? Such considerations have not been fully implemented in clinical practice, where many patients without risk factors received chemotherapy and vice versa. The most commonly held belief is that dMMR/MSI-H is a predictor of decreased benefit from adjuvant chemotherapy when a fluoropyrimidine is used alone in patients with stage II disease. However, in some studies the data do not agree [4]. Therefore, the primary tumor location will be a powerful addition to MMR status and risk factors, and provide a better indication of which patients should be selected for adjuvant chemotherapy.

Neither clinically precise guidance nor practicability of adjuvant chemotherapy is guaranteed if the MMR status, risk factors, and primary tumor location are not considered together. Therefore, a combination of the two methods is needed. In the non-chemotherapy cohort, the combination of pMMR and RCC, the two subgroups with poor prognosis respectively, had the poorest prognosis. Interesting, only this group attained benefit from chemotherapy. At the same time, we found that for patients who received chemotherapy, both the chemotherapy regimens and number of risk factors were similar across all the four groups. Li [42] found that the combination of MMR status and tumor location helped to stratify CRC patients, where RCC patients with dMMR had a higher OS than those with pMMR, as was similarly observed in LCC patients. In contrast, rectal cancer patients with dMMR had a lower OS than those with pMMR. However, this study did not investigate the use of chemotherapy.

Our findings require further investigation and validation since this was a retrospective study. The effects of treatments after relapse or metastasis, which inevitably impact upon OS, were unclear. However, we believe it is welcoming that the DFS in most conditions also showed significant accordance with OS. The judgment of MMR status depended only on the immunohistochemistry results and did not distinguish between the deficiency subtypes. Therefore, it was not possible to accurately determine MSS, MSI-low, and MSI-H. Fortunately, MMR status has been shown to be highly consistent with MSI status [43]. Finally, one other limitation of this study was the small number of patients, especially the dMMR subgroup. All of these limitations need to be overcome in future studies.

In summary, for the first time we have demonstrated that when combined with MMR status, the primary tumor location provides superiority in patient selection for pMMR /RCC patients who require adjuvant chemotherapy. This has implications for risk factors recommended by the NCCN guideline in assisting the selection of adjuvant chemotherapy for CRC patients with stage II disease.

## Abbreviations

MMR: (mismatch repair)
LCC: (left-sided colon cancer)
RCC: (right-sided colon cancer)
OS: (overall survival)
DFS: (Disease-free survival)
DMMR: (mismatch repair-deficient)
MMR: (mismatch repair-proficient)
NCCN: (National Comprehensive Cancer Network)
LV: (leucovorin)
5-FU/LV: (5-Fluorouracil/Leucovorin)
FOLFOX: (5-Fluorouracil+oxaliplatin+Leucovorin)
CapeOX: (Oxaliplatin+Capecitabine)
FLOX: (5-Fluorouracil+oxaliplatin+Leucovorin)
IFL: (irinoteca
mCRC: (metastatic colorectal cancer)
ALB: (albumin)
TLN: (Total lymph node)
